# Molecular and Cellular Regulation of Toll-Like Receptor-4 Activity Induced by Lipopolysaccharide Ligands

**DOI:** 10.3389/fimmu.2014.00473

**Published:** 2014-10-06

**Authors:** Ardiyanto Liaunardy-Jopeace, Nicholas J. Gay

**Affiliations:** ^1^Department of Biochemistry, University of Cambridge, Cambridge, UK

**Keywords:** toll-like receptor 4, trafficking, sensitization, allergens, endogenous ligands

## Abstract

As well as being the primary signaling receptor for bacterial endotoxin or lipopolysaccharide Toll-like receptor-4 function is modulated by numerous factors not only in the context of microbial pathogenesis but also autoimmune and allergic diseases. TLR4 is subject to multiple levels of endogenous control and regulation from biosynthesis and trafficking to signal transduction and degradation. On the other hand regulation of TLR4 activity breaks down during Gram −ve sepsis leading to systemic damage, multi organ failure, and death. In this article, we review how TLR4 traffics from the early secretory pathway, the cis/trans Golgi to the cell surface and endolysosomal compartments. We will present evidence about how these processes influence signaling and can potentially lead to increased sensitivity to ligand-dependent activation as well as ligand-independent constitutive activation that may contribute to pathogenesis in sepsis. We will also discuss how sustained signaling may be coupled to endocytosis and consider the potential molecular mechanisms of immuno-modulators that modify TLR4 signaling function including the cat allergen FelD1 and endogenous protein ligands such as the extracellular matrix protein tenascin C and calprotectin (MRP8/14).

## Introduction

Due to its importance in host innate immune response against infection, as well as in pathogenesis of autoimmune diseases and chronic inflammatory conditions, TLR4 signaling activity is subject to complex regulation (1). TLR4 activates two distinct pathways originating from different cellular locations, the cell surface, and the endosome. This results either in inflammatory responses mediated by the adaptor MyD88 and transcription factor NFκB or anti-viral signaling responses transduced by TRAM/TRIF and IRF3 (2). It is thus critical to ensure that signals are appropriately activated at the right place and the right time, and are terminated when no longer required. The roles of accessory and adaptor molecules in the regulation of TLR4 signaling from biosynthesis to activation and eventually to degradation have been subject to intensive study (Table 1). CD14 and MD2, for example, are important for recognition and delivery of ligand LPS to receptor at the cell surface, whereas the cytosolic TIR domain-containing adaptors determine which pathway is activated. These two molecules, however, have additional roles in the trafficking and localization of TLR4 receptor, before and after LPS stimulation, which will be discussed in Sections “Biosynthesis and Localization of TLR4” and “Vesicular Trafficking and Signaling of TLR4.” Last year the Nobel Prize in Physiology or Medicine was awarded for molecular studies of vesicular trafficking, recognizing the importance of these fundamental processes for the biosynthesis and trafficking of secreted proteins and for cellular regulation. Dysregulation of these pathways can result in over-sensitization TLR4 responses. In addition, several non-canonical activators of TLR4, such as cat allergen FelD1, have been described recently to induce sensitization of the receptor. Here, we review recent advances that shed light on the mechanisms that regulate TLR4 at the molecular and cellular level with an emphasis on the role of protein secretory pathways.

**Table 1:** **Accessory molecules that regulate TLR4 signaling activity**.

Accessory molecules	Roles in the regulation of TLR4 signaling	Reference
PRAT4A	TLR4 folding in the ER	(3, 4)
gp96	TLR4 folding in the ER	(5)
MD2	Correct glycosylation of TLR4, and accessory molecule for LPS recognition	(6–9)
CD14	Co-receptor for LPS on the cell surface, and promotes LPS-induced endocytosis of the activated receptor	(10–12)
TMED7	Trafficking of TLR4 to the cell surface/to the late endosome^a^	(13, 14)
Rab10	Trafficking of TLR4 to the cell surface	(15)
Rab11a	Trafficking of TLR4 from endocytic recycling compartment to *E. coli*-containing phagosome	(16)
Rab7b	Degradation of TLR4 in the lysosome	(17)
MyD88	Adaptor molecule for TLR4 signaling transduction inside the cell	(18–20)
Mal	Adaptor molecule for TLR4 signaling transduction inside the cell	(21, 22)
TRIF	Adaptor molecule for TLR4 signaling transduction inside the cell	(23, 24)
TRAM	Adaptor molecule for TLR4 signaling transduction inside the cell	(25, 26)
SARM	Negative regulator of TLR4 signaling	(27–29)
CD11b	Positive regulator of TLR4 signaling	(30)
PLC Cγ-2	Promotes LPS-induced endocytosis of activated TLR4	(31)

*A non-exhaustive list showing molecules that play roles in regulating the activity of TLR4 signaling that ranges from the biosynthesis stage of the receptor in the early secretory pathway to the degradation of the activated receptor in the lysosomes. Only references that have direct connections to TLR4 are listed here*.

*^a^Currently, there are two opposing conclusions from two independent studies on the roles of TMED7 in the signaling of TLR4*.

## Biosynthesis and Localization of TLR4

Two chaperone molecules glycoprotein (gp) 96 and protein associated with TLR4 (PRAT4A) are required for the correct folding of TLR4, and other TLRs, in the ER (3–5, 32). Both chaperones interact with TLR4 in the ER and depletion of either molecule results in reduced cell surface expression of TLR4 and hence lower activity in response to LPS stimulation [Reviewed in Ref. (33)]. In addition to its role as a co-receptor for LPS on the cell surface, MD2 is also required for correct glycosylation of TLR4 during its biosynthesis. MD2 associates with the nascent TLR4 in the ER, possibly assisted by the chaperones, and at least in embryonic fibroblasts from MD2^−/−^ mice, TLR4 is not transported to the cell surface but accumulated in the Golgi (6). This is because MD2 is required for correct N-glycosylation of TLR4 that enables the mature receptor complex to be trafficked to the cell surface (7, 8). Overall, correct post-translational modification of TLR4 in the ER, especially the addition of mannosyl N-linked glycans, is important for cell surface localization of the mature receptor, which is crucial for ligand-dependent signaling activity.

## Vesicular Trafficking and Signaling of TLR4

### Vesicular trafficking of TLR4 from the ER to the cell surface

**Figure 1:**
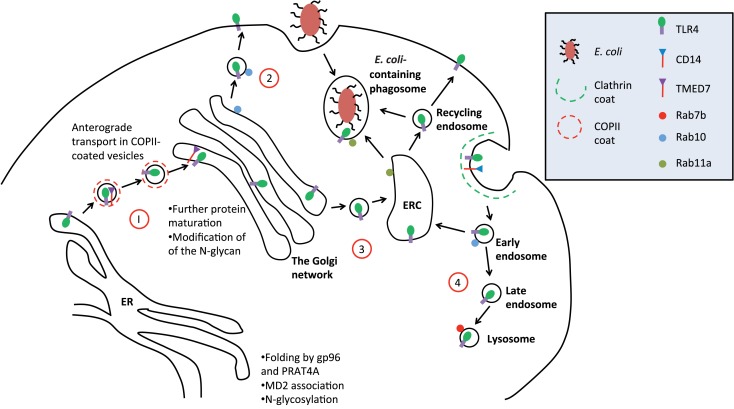
**Trafficking of TLR4**. A simplified schematic of TLR4 trafficking in the early secretory and endocytic pathways. (1) Upon translation, folding, and glycosylation of the protein in the ER, nascent TLR4 is recognized by TMED7 cargo receptor to be trafficked anterogradely in COP II-coated vesicles toward the Golgi complex (13). (2) Following maturation within the Golgi complex, mature TLR4, along with MD2 (not shown in diagram), is transported to the cell surface via vesicular trafficking that is Rab10 dependent (15). (3) Alternatively, mature TLR4 can be translocated to endosomal recycling compartment (ERC) where it forms a distinct intracellular pool of receptors that can recognize phagocytosed Gram-negative bacteria such as *E. coli* (16). From here, activated TLR4 can mount innate immune responses intracellularly independent of the cell surface receptor. It is likely that the ERC also act as a recycling organelle for new and old receptors back to the cell surface to resensitize the cell. (4) Finally, upon receptor activation on the cell surface, receptor is endocytosed into early endosome where TRIF/TRAM pathway is initiated. Eventually, the early endosome matures into late endosome fuse with the lysosome where the receptor will be degraded to terminate the signaling. This process is Rab7b-dependent.

In the ER, correctly folded cargo for anterograde transport to the Golgi is selected for packaging into COPII-coated vesicles (Figure 1). Selection of folded glycoprotein cargo such as TLR4 seems to require firstly the presence of a specific octa-mannosyl N-linked glycan moiety (34). Other patterns of mannosylation direct glycoproteins to the ER quality control compartment (ERQC) for refolding or ERAD mediated destruction. A second requirement is a cytosolic diphenylalanine motif that acts as a signal for the assembly of the COPII coat (35). Many secreted soluble and transmembrane proteins do not have this motif but instead require adaptor proteins for packaging into COP II vesicles and transport to the Golgi.

A recent study shows that one such adaptor TMED7 is necessary for anterograde trafficking of TLR4 to the cell surface (13). TMED7 is a type I membrane protein with a N-terminal luminal GOLD domain followed by a coiled-coil dimerization sequence, a single transmembrane helix, and a short cytoplasmic tail that contains a diphenylalanine motif (36, 37). Humans have 9 TMED7 paralogs and family members play important roles in trafficking and membrane homeostasis as studied in yeast model organisms. The yeast ortholog of TMED2 or Emp24p functions in the secretion of glycoproteins invertase and GPI-anchored Gas1p (38). Other roles of the TMED family range from maintaining the structural integrity of the Golgi (39, 40), retention of ER-resident proteins (41), and unfolded protein responses (42) to mouse embryonic development (43). TMED7 binds stably to the TLR4 ectodomain an interaction that requires the GOLD and coiled-coil domains. Full length TMED7 concentrates in the cis-golgi but removal of the diphenylalanine motif causes it to redistribute in the endomembrane system. The truncated form of TMED7 also causes constitutive activation of TLR4, perhaps because it cannot be transported to the Golgi but accumulates in the ER (13). This finding suggests that under conditions of cellular stress such as might be found in sepsis the production of inflammatory mediators by TLR4 may be independent of LPS. Thus, therapies that target receptor homo-dimerization, a key step in the activation pathway of TLR4, may be more effective than LPS antagonists.

It remains unclear how TMED7 and TLR4 interact with each other in the ER and how they dissociate during trafficking to cell surface. On the other hand, it is known that the small GTPase Rab10 co-localizes with TLR4 in the Golgi and enhances TLR4 signaling activity by increasing the rate of TLR4 trafficking to the cell surface from the Golgi when cells are stimulated with LPS (15). Rab10, a member of the Ras family, is likely to assist this process by positively regulating vesicle formation and fusion with the target compartments. Rab10 expression is elevated in dendritic cells and macrophages after LPS stimulation, which acts as a positive feedback to ensure more TLR4 receptors are translocated to the cell surface so that cells remain responsive to LPS. However, Rab10 is a soluble cytosolic protein and cannot itself select cargo for vesicular trafficking to the cell surface (44). Thus, it is likely that a transmembrane trafficking adaptor is required to act as a specific cargo receptor to couple Rab10 to TLR4, a role that could also be carried out by TMED7. Another family member, the mammalian TMED2, and its cargo molecule, the 7-TM protease-activated receptor 2 (PAR2) may provide a relevant analogy. Like TMED7 and TLR4, TMED2 forms complexes with its cargo PAR2 that require the GOLD and dimerization motifs of the adaptor and an extracellular loop of the receptor (45). In order for PAR2 to traffic to the surface, TMED2 is dissociated from the complex by the activation of Arf1, another member of the small GTPase superfamily. Interestingly, LPS stimulation leads to a significant reduction in the amount of TMED7/TLR4 complexes present in the cell consistent with the idea that Rab10 plays a similar role in trafficking to that fulfilled by Arf1 in the case of PAR2.

### Endocytic trafficking of TLR4

Activation of TLR4 by LPS appears to be coupled to internalization and this depletes the cell surface TLR4 receptors, which could cause cells to become desensitized to the stimuli. Indeed a study has shown that internalization of TLR4 in the absence of LPS, induced by an anti-CD14 antibody on the cell surface, reduced LPS responsiveness in human primary monocytes and THP-1 cells (46). Constant translocation of TLR4 from the Golgi to the cell surface is therefore required to replenish the level of cell surface TLR4 in the presence of LPS and to allow a sustained signaling response (47). Endocytosis of the activated receptor complex from the cell surface into the early endosome has two important consequences: the activation of the TRAM/TRIF pathway and the termination of the signaling (12). Activation on the cell surface and endocytosis are coupled by CD14 and TRAM and the endocytosis process is clathrin and dynamin-dependent (10, 48, 49).

## Sensitization of TLR4 Responses by Allergens, Metals, and Endogenous Ligands

The activation of innate pattern recognition receptors such as TLR4 is required to initiate both innate and adaptive immune responses. These recognition and signaling processes also play a central role in the development of inflammatory and autoimmune diseases such as rheumatoid arthritis, asthma, and septic shock (50). In the case of TLR4, the identification of bonafide ligands and agonists has been hindered due to the ubiquity of LPS in the environment causing contamination of ligand preparation. Nevertheless a consensus is emerging that direct ligands that can bind the receptor and induce dimerization are limited to LPS, nickel and other divalent transition metals, the synthetic cationic lipid, di-C14 amidine, and paclitaxel (1, 51, 52, Lonez et al., submitted). There are, however, a number of other molecules that enhance the activity of TLR4 but may not be direct ligands of the receptor. These include allergens such as FelD1, high-mobility group protein B (HMGB), tenascin, proteoglycans, calprotectin [also known as the cytosolic myeloid related proteins (MRP) 8 and 14 and S100A8/9] (53–57).

The lipid A moiety of LPS is sufficient to activate TLR4. The acyl chains of immunostimulatory LPS intercalate into the β-sandwich fold of MD-2. One of the six fatty acyl chains is partially exposed on the surface of MD-2, creating a hydrophobic patch that can form an interface with another TLR4/MD-2 heterodimer (58). Ionic interactions mediated by the glucosamine phosphate backbone of LPS further stabilize this MD-2-TLR4 interface and promote the formation of a secondary homo-dimerization site between the lateral surfaces of the receptor’s leucine rich repeat (LRR) solenoids (59). This leads to the assembly of an “M” shaped heterotetramer that positions the C-termini of the LRR solenoids in close proximity, allowing the cytosolic TIR domains of the receptor to dimerize. By contrast, divalent metal ions such as Ni^2+^ and Co^2+^ that can induce contact dermatitis in humans act by binding to specific histidine residues in the secondary receptor homo-dimerization site, again leading to assembly of the active heterotetramer (60). A recent study has identified another direct mechanism for TLR4 activation. In this case the cationic, di-acyl lipid di-C14 amidine activates TLR4 by a mechanism that is independent of MD-2. Instead di-C14 amidine is predicted to bind to a hydrophobic crevice in the receptor homo-dimerization site, stabilizing the formation of this interface.

By contrast to the activators described above another group of immuno-modulators do not directly induce assembly of the activated TLR4/MD-2 heterotetramer. The major cat allergen, the dander protein FelD1, enhances TLR4 signaling by about 10-fold but does not bind to TLR4/MD-2 (53). Instead, it is likely that FelD1 can sequester environmental LPS and other lipid TLR agonists. Thus, dander proteins loaded with environmentally derived PAMPs may associate with cell membranes, facilitating lipid presentation, and transfer to accessory molecules such as CD14 or directly to receptor complexes. Alternatively, FelD1 may promote greater clustering of TLR4-bearing lipid rafts, leading to increased receptor activation. DerP2 from the dust mite and Canf6 from dog, two allergens that are structurally distinct from FelD1, also enhance TLR4 activity suggesting that this may be a common feature of allergen action. It is possible that this lipid transfer or raft stabilization mechanism may underlie the properties of endogenous TLR activators as well. For example, the Mrp8/Mrp14 protein complex calprotectin enhances LPS activation of TLR4 when presented extracellularly. Mrp8/14 are calcium binding EF-hand proteins and they associate with lipid raft structures (61). A third class of endogenous activator is the large extracellular matrix protein tenascin C. Tenascin C is induced by tissue damage and the C-terminal fibrinogen globe (FBG) module causes activation of TLR4 in chronic inflammatory disease such as rheumatoid arthritis (56). Attempts to show direct binding of FBG to TLR4/MD-2 have not been successful suggesting an indirect mode of action for FBG.

## Conclusion

In conclusion, TLR4 responsiveness to its bonafide ligand, LPS, can be further regulated by its cellular localization and the clustering effect induced by immuno-modulatory molecules such as the cat allergen FelD1. The regulation of the recycling and trafficking of many membrane receptors, especially TLR4, is dynamic and involves cargo receptors and small GTPase molecules. This provide an additional control to receptor signaling activity in addition to gene expression control, post-translational modifications prior to the arrival of signal, and recruitment of various adaptor molecules and kinases downstream of the receptor activation by ligands. Compartmentalization is particularly important for TLR4 as it dictates which pathway is activated upon receptor stimulation (16). These studies provide insight into a different mode of receptor regulation through membrane-bound vesicular trafficking. Allergens and endogenous activators may display a second non-canonical mode of regulation of TLR4 by functioning as lipid binding proteins or membrane microdomain stabilizers (31, 62).

## Conflict of Interest Statement

The authors declare that the research was conducted in the absence of any commercial or financial relationships that could be construed as a potential conflict of interest.
